# Integrating Comprehensive Rehabilitation Care to Multimorbidity Approach: A Challenge for the Chilean Public Health System

**DOI:** 10.5334/ijic.7697

**Published:** 2024-02-01

**Authors:** Paula Zamorano, Fernanda Calvo, Ricardo Banda, Javiera Fuentes, Clara Molina, Elena Medina, Marcela Gonzalez-Madrid

**Affiliations:** 1 Innovación ANCORA UC, Facultad de Medicina, Pontificia Universidad Católica de Chile, Santiago, Chile; 2 Departamento de Ciencias de la Salud, Pontificia Universidad Católica de Chile, Santiago, Chile; 3 Departamento de la Ciencia de la Ocupación y Terapia Ocupacional, Facultad de Medicina, Universidad de Chile, Santiago, Chile; 4 School of Health Professions Education, Faculty of Health, Medicine and Health Sciences Maastricht University, Maastricht, Netherlands; 5 Red de Salud UCChristus, Unidad de Rehabilitación, Chile; 6 Departamento de Salud del Adulto y Senescente, Escuela de Enfermería, Pontificia Universidad Católica de Chile, Santiago, Chile

**Keywords:** integrated care, rehabilitation, multimorbidity care, interdisciplinarity, Cuidado centrado en la persona, rehabilitación, multimorbilidad, interdisciplina

## Abstract

The multimorbidity approach involves promotional and preventive strategies. The demand for rehabilitation services has grown exponentially in recent years, leading to the urgency of rethinking care delivery. In Chile, there are laws, programs, and guidelines that, from their theoretical basis, include a person-centered care focus. But in real practice, multiple barriers trigger important fragmentation of care. In response, a new strategy has been proposed to answer whether comprehensive rehabilitation care based on multimorbidity positively impacts the health system performance, people’s functionality, and quality of life, which will be implemented as a pilot study with a national scale-up focus.

## Abstract (es)

El enfoque de multimorbilidad implica estrategias promocionales y preventivas. La demanda de servicios de rehabilitación ha crecido exponencialmente en los últimos años, lo que ha llevado a la urgencia de repensar la organización y entrega de prestaciones. En Chile existen leyes, programas y lineamientos que, desde su base teórica, incluyen un enfoque de atención centrado en la persona. Pero en la práctica real, múltiples barreras generan una importante fragmentación de la atención. En respuesta, se ha propuesto una nueva estrategia para responder si una rehabilitación integral basada en el enfoque de la multimorbilidad impacta positivamente en el desempeño del sistema de salud, la funcionalidad de las personas y la calidad de vida, que se implementará como un estudio piloto con un enfoque de escalamiento nacional.

## Background

Multimorbidity prevalence has increased during the last few years, challenging health systems worldwide, especially ambulatory care [1]. The impact on the health system, the family environment and society reveal the urgency to reorganize and rethink how health care is delivered for chronic patients [2]. According to data from the latest National Health Survey in Chile in 2016, more than 70% of the adult population has multimorbidity [3]. The public health system response for chronic care at primary health care (PHC) is organized on a single diagnostic approach with vertical programs (cardiovascular, respiratory, mental health, musculoskeletal, disability, elderly, etc.) and laws (Ley 19.966 Health explicit Guarantees among others) that fragments care, affecting efficiency and efficacy.

**Figure 1:**
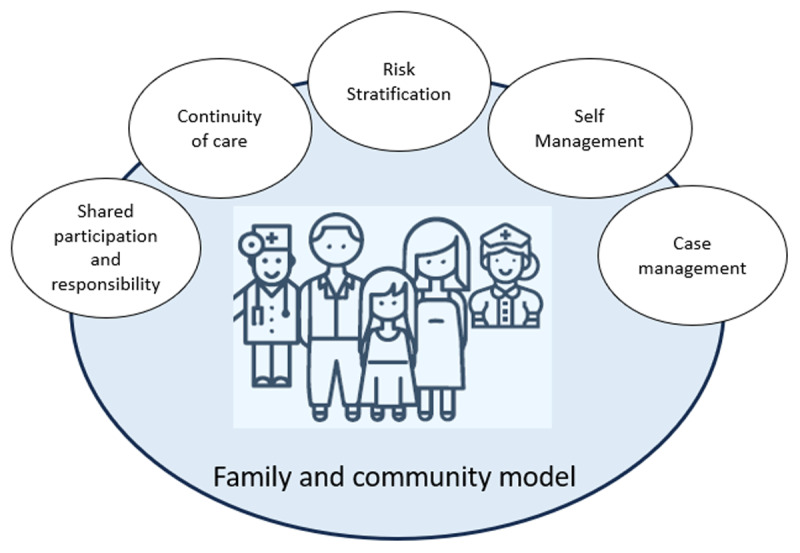
Multimorbidity patient-centered care model [4].

Therefore, in 2017 a Multimorbidity Patient-Centered Care Model [4] (Figure 1) began its pilot implementation in the public health system, mainly in PHC. The model was based on the Family and Community model already existing at PHC and other core elements such as case management, self-management, and risk stratification. This strategy reorganized the existing clinical services according to each patient’s multimorbidity risk. High-risk patients receive more frequent care based on case management and transition care. Moderate and low-risk patients received care from the primary care team but were less frequent and focused on improving self-management skills. During the four years of piloting, more than 22,000 adult patients were intervened, and positive results were shown in health services utilization and mortality risk [5,6,7]. Given its success and the experience of other local initiatives, the government began scaling up a similar strategy in 2020 [8], covering almost the entire national territory.

Chile has pioneered in Latin America in transforming adult chronic care from a single diagnostic approach to a multimorbidity patient-centered approach [8,9]. However, the scaling-up implementation of this complex change has been mainly focused on chronic care at primary care centers. Thus, integrating other allied prevention services, such as rehabilitation care, is still challenging.

### Rehabilitation services in Chile

During the last decades, Chile has made great progress in reducing mortality rates and other indicators favoring its population’s life expectancy, such as the MACEP. However, our country hasn’t provided the necessary development to respond to the rehabilitation needs of a population that survives with major disease burdens and higher health spending. As a result, the prevalence of disability in the adult population is 16.7% [10] and the demand for rehabilitation services has increased explosively over the past few decades, adding pressure to significant limitations in access and infrastructure. This reality became evident during the COVID-19 pandemic. It has been estimated in secondary sources that the gap in the public system in human resources of rehabilitation is close to 45% if compared to the international standard and that waiting times are close to 180 days for rehabilitation therapy.

Rehabilitation in adult patients comprises comprehensive and integrated care from its theoretical scientific basis [11,12]. In Chile, government orientations guide care delivery under this paradigm. However, the single diagnostic approach prevails in real practice, leaving aside the multimorbidity focus [13,14,15,16]. For example, prioritization is mainly based on individual health problems of the GES Plan (Health Explicit Guarantees) [17] rather than risk stratification or patient complexity by providing standard numbered sessions. Self-management activities are offered for certain groups of diseases [13,18,19] rather than multimorbidity clusters. The health professional vertically assigns individualized care plans and treatment goals rather than establishing them on shared decision-making, despite most rehabilitation centers having a rehabilitation team composed of physio and occupational therapists [12,20]. Finally, the scarce capacity [3] and performance incentives fragment the continuity and coordination of care, triggering waiting lists, medical leaves, and extended waiting times for care [21,22,23]. Therefore, integrating rehabilitation care into the multimorbidity approach would probably improve patient outcomes, quality of life and health systems performance, which is the aim of this commentary and innovation on the field from the Pontificia Universidad Católica de Chile (PUC).

This article aims to comment on the challenge of integrating highly demanded allied primary and secondary services such as rehabilitation to the multimorbidity patient-centered approach.

## Multimorbidity comprehensive rehabilitation care strategy

**Figure 2:**
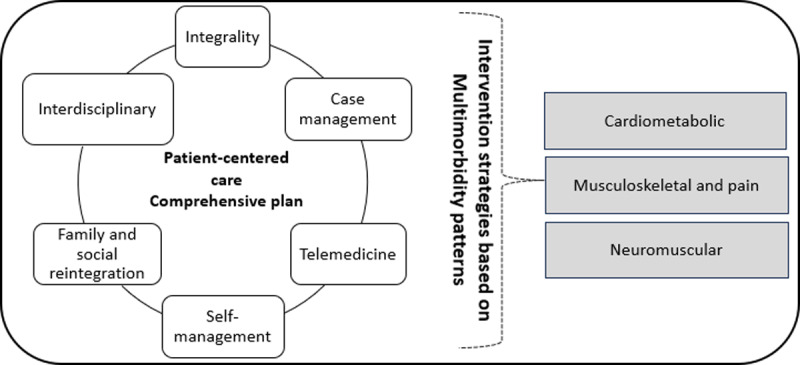
Comprehensive rehabilitation care strategy.

In 2022, the PUC and its allied healthcare academics responded by funding the design of an innovative, interdisciplinary *Multimorbidity comprehensive rehabilitation care strategy* (Figure 2), which will be implemented in more than 400 square meters of the rehabilitation service in the Centro de Innovación en Salud ANCORA San Francisco located in the municipality of Puente Alto, a multidimensional vulnerable territory of the metropolitan region. This strategy will be organized and commissioned in early 2024 by the PUC and expects to benefit an estimated population of 265,000 people with disabilities from this territory, increasing the supply to adult and pediatric patients.

The strategy is based on integrated care, case management, continuity of care, family and social reintegration, telemedicine and interdisciplinarity. Its objective is to deliver innovative and interdisciplinary rehabilitation services, ensure access and continuity of care, and promote self-management in adults with multimorbidity through interdisciplinary care delivery of physiotherapy, occupational therapy, speech therapy, nutrition, and nursing. This rehabilitation service will be continuously evaluated to consolidate a model that can be replicated at a country level and thus significantly improve rehabilitation services in our public health system. In addition, to develop a clinical-academic center which includes interdisciplinarity, innovation and reflection as differentiating aspects of the learning process of students of the allied health careers.

Clinical services start with a comprehensive evaluation where the patients’ level of functionality is assessed, and treatment goals are agreed upon to define the individualized interdisciplinary treatment plan that will guide the different professionals’ interventions. Then, the organization of the clinical activities and services is based on multimorbidity patterns such as neuromuscular, cardiometabolic and musculoskeletal, prioritizing the patient’s time, where sequential or double care, face-to-face or remote, will be a core aspect of this service.

The practice of daily living activities in a specialized area, self-management workshops and parallel services for patient carers are offered to each patient according to their needs and preferences to improve self-management skills for patients with chronic non-communicable diseases. Services will be monitored through case management, which, in addition to focusing care on patients’ needs, will ensure continuity and transition with their primary care team, either from the ANCORA or public networks.

For example, an adult patient with five chronic diseases who suffers from osteoarthritis will receive a comprehensive evaluation where the functional capacity will be measured along with other standard evaluations. Then, with the rehabilitation professional, review the services available for musculoskeletal multimorbidity patterns such as interdisciplinary therapy, self-management workshops based on pain neuroscience education, educational asynchronous support, and practice in the daily living area. They will establish individualized treatment goals and define which interventions/services are the most appropriate for the patient’s needs. Then, with the case manager’s support, continuity of care will be monitored, and at the end of the treatment, a referral will be provided for the primary care team.

Finally, implementing the strategy implies generating important cultural, organizational, and structural changes that should be properly addressed, incorporating change management activities and training in communication and motivational skills to facilitate adherence to more comprehensive and multidisciplinary care.

## Conclusion and Recommendations

This innovation takes a step forward, incorporating associated centers and allied secondary prevention services into this multimorbidity paradigm shift. Through this implementation, we hope to answer whether comprehensive rehabilitation care based on multimorbidity generates positive results in the health system performance, people’s functionality, chronic self-management, and quality of life. Implementing the proposed intervention will provide lessons from real practice on reorganizing allied primary care preventive services that would provide valuable insights for decision-makers driving the national scale-up of the multimorbidity strategy. Furthermore, the center will strongly contribute to training health professionals with a comprehensive perspective which is fundamental for successful health care.
